# Supplementary material for: Comparative Proteomics Analysis between Maize and Sorghum Uncovers Important Proteins and Metabolic Pathways Mediating Drought Tolerance
Source: Life (Basel). 2023 Jan 6;13(1):170. doi: 10.3390/life13010170 (PMC9863747; doi:10.3390/life13010170)

Figure S1 One-dimensional leaf proteins of *Zea mays* (a) and *Sorghum bicolor* (b) from five biological replicates. Total soluble protein (10 µg) of leaf tissue for each plant species was loaded onto 12 % SDS-PAGE gels. Lane M is the molecular weight marker; Lanes 1-10 show protein profiles from five independent biological replicate extractions for the leaf tissue for each plant species. Well-watered (WW), water deficit (WD).

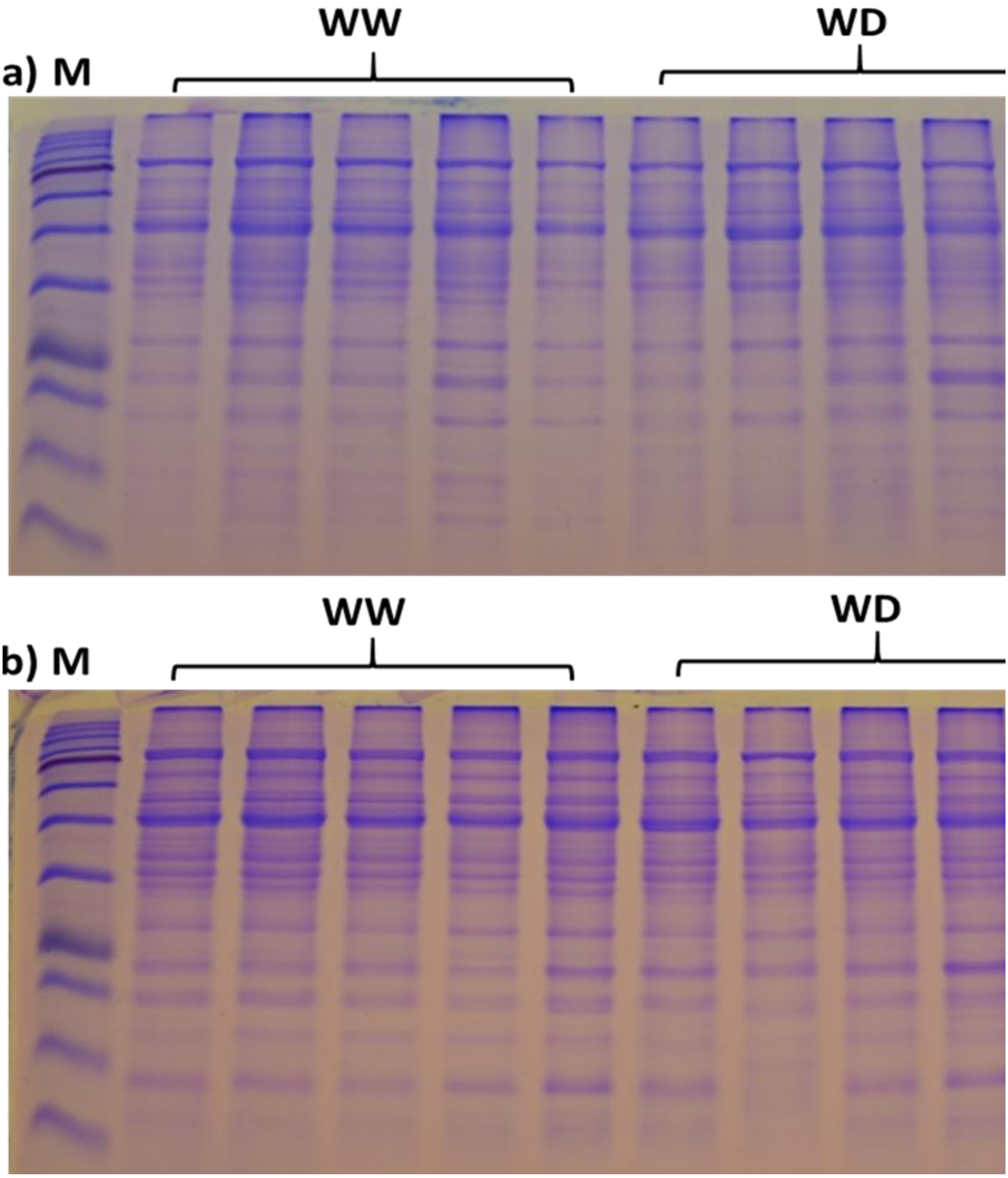

Supplement: Supplementary file 1 [file life-13-00170-s001.zip › Supplementary Figure S1.pdf]
