# Supplementary material for: Comparative Proteomics Analysis between Maize and Sorghum Uncovers Important Proteins and Metabolic Pathways Mediating Drought Tolerance
Source: Life (Basel). 2023 Jan 6;13(1):170. doi: 10.3390/life13010170 (PMC9863747; doi:10.3390/life13010170)
Supplement: Supplementary file 1 [file life-13-00170-s001.zip › Supplementary Table S1.pdf]

Table S1 Details of LC-MS/MS data acquisition

**Full Scan**	
Resolution	70,000 (@ *m/z* 200)
AGC target value	1e6
Scan range	350-2000 m/z
Maximal injection time (ms)	250
**Data-dependent MS/MS**	
Inclusion	Off
Number of MS/MS scans produced	1,000,863
Resolution	17,500 (@ *m/z* 200)
AGC target value	1e5
Maximal injection time (ms)	50
Loop Count	5
Isolation window width (Da)	2
NCE (%)	27
**Data-dependent Settings**	
Underfill ratio (%)	1
Charge exclusion	1, 7,8, >8
Peptide match	Preferred
Exclusion isotopes	On
Dynamic exclusion (s)	60
